# Information use and plasticity in the reproductive decisions of malaria parasites

**DOI:** 10.1186/1475-2875-13-115

**Published:** 2014-03-26

**Authors:** Lucy M Carter, Petra Schneider, Sarah E Reece

**Affiliations:** 1 Institute of Evolutionary Biology, School of Biological Sciences, Ashworth Laboratories, University of Edinburgh, Edinburgh, UK; 2 Centre for Immunity, Infection & Evolution, Institutes of Evolution, Immunology and Infection Research, School of Biological Sciences, Ashworth Laboratories, University of Edinburgh, Edinburgh, UK

**Keywords:** Transmission, Gametocyte investment, Conversion rate, Sex ratio, Host-parasite interactions, Competition, Phenotypic plasticity

## Abstract

**Background:**

Investment in the production of transmissible stages (gametocytes) and their sex ratio are malaria parasite traits that underpin mosquito infectivity and are therefore central to epidemiology. Malaria parasites adjust their levels of investment into gametocytes and sex ratio in response to changes in the in-host environment (including red blood cell resource availability, host immune responses, competition from con-specific genotypes in mixed infections, and drug treatment). This plasticity appears to be adaptive (strategic) because parasites prioritize investment (in sexual *versus* asexual stages and male *versus* female stages) in manners predicted to maximize fitness. However, the information, or ‘cues’ that parasites use to detect environmental changes and make appropriate decisions about investment into gametocytes and their sex ratio are unknown.

**Methods:**

Single genotype *Plasmodium chabaudi* infections were exposed to ‘cue’ treatments consisting of intact or lysed uninfected red blood cells, lysed parasitized RBCs of the same clone or an unrelated clone, and an unmanipulated control. Infection dynamics (proportion of reticulocytes, red blood cell and asexual stage parasite densities) were monitored, and changes in gametocyte investment and sex ratio in response to cue treatments, applied either pre- or post-peak of infection were examined.

**Results and conclusions:**

A significant reduction in gametocyte density was observed in response to the presence of lysed parasite material and a borderline significant increase in sex ratio (proportion of male gametocytes) upon exposure to lysed red blood cells (both uninfected and infected) was observed. Furthermore, the changes in gametocyte density and sex ratio in response to these cues depend on the age of infection. Demonstrating that variation in gametocyte investment and sex ratio observed during infections are a result of parasite strategies (rather than the footprint of host physiology), provides a foundation to investigate the fitness consequences of plasticity and explore whether drugs could be developed to trick parasites into making suboptimal decisions.

## Background

Malaria parasites proliferate in the blood through cycles of asexual replication, but every cell cycle a small proportion of progeny commit to developing into male and female gametocytes (which do not replicate in the host) [1-4]. This means that, like all sexually reproducing organisms, malaria parasites face resource allocation trade-offs between survival and reproduction and between producing males and females [5-8]. Specifically, every cell cycle parasites make decisions about how much to invest in gametocytes (which are essential for reproduction and transmission) *versus* asexuals (which are essential for in-host survival) and in males *versus* females. These decisions are sensitive to variation in the in-host environment [9,10].

Extensive variation in gametocyte investment (also known as the ‘conversion rate’ or ‘reproductive effort’) and sex allocation (proportion of male gametocytes) of *Plasmodium spp.* has been observed across different species, strains, and during infections [10-18]. Understanding variation in gametocyte investment and sex ratio (collectively referred to as ‘reproductive strategies’) is important because they are key fitness-determining traits, shaping survival within hosts and the success of transmission to new hosts [19-21]. Experiments using rodent malaria parasites *in vivo* and *Plasmodium falciparum in vitro* suggest that parasites alter investment in gametocytes and their sex ratio in response to: changes in red blood cell (RBC) resource availability [12,18,22-24], host derived transmission blocking immune (TBI) responses [25-28], competition from con-specific genotypes in mixed infections [16,25,29,30] and, drug treatment [11,13,14,31-35]. Observational data from natural infections also suggests that *P. falciparum* sex ratios and gametocyte investment differ between single and mixed infections and are altered in response to variation in RBC density [36].

Evolutionary theory offers explanations for why parasites adjust their reproductive strategies in response to the changing environmental conditions encountered in the host [10,20,37-39]. For example, parasites increase gametocyte investment in response to anaemia, reticulocytes and exposure to sub-lethal anti-malarial therapy [11,13,18,23,32,33,40,41]. This has been interpreted as a strategy of ‘terminal investment’ during extreme stress [42]: investing heavily in gametocytes maximizes transmission potential in a situation likely to be lethal (e.g., before the infection is cleared or the host dies) [11,13,32]. However, recent evolutionary theory predicts that this may be an oversimplification and that less severe stress induces parasites to reduce investment, as a strategy of ‘reproductive restraint’ [39]. Reproductive restraint is predicted to facilitate in-host survival and therefore future transmission opportunities [39]. Empirical work supports these predictions, revealing that when parasites experience competitive suppression, RBC limitation, and low doses of anti-malarial drugs, they reduce gametocyte investment [14,16,30]. The sex allocation decisions of parasites are sensitive to many of the same factors as gametocyte investment. For example, different sex ratios bring the highest fitness returns in single- *versus* mixed-genotype infections [6,25,43-46] and when hosts are mounting immune responses that differentially affect male and female gametocytes [47]. Experiments with *Plasmodium chabaudi* reveal that sex ratios are precisely allocated according to the number of co-infecting genotypes and their relative representation within a mixed-genotype infection [25]. Therefore, sex ratio data suggest that parasites can determine the genetic diversity of their infections and measure the number (or replication rate) of asexual stages belonging to their genotype [25].

Whilst evolutionary theory can explain why parasites adjust investment into gametocytes and their sex ratio, it does not explain how they do so. Whether parasites identify and respond to individual factors (e.g., RBC density and age structure, the presence of competing parasites and the dose of drugs), or the overall impact the environment has on their proliferation rate (i.e., ‘state’) is not known [21]. A further complication is that the in-host environment is complex and many factors change simultaneously. For example, both anaemia and immunity develop as parasite number increases [26,48], competition in mixed infections brings RBC limitation and suppresses asexual proliferation [9,49-51], and different drugs kill parasites in dose-dependent ways and can alter anaemia [52]. For the parasite, more accurate information may be obtained from directly measuring individual environmental factors, but measuring changes in overall state may be the most efficient strategy, as it does not require the assimilation of information from multiple environmental variables that could elicit contradictory parasite responses [21].

The experiments presented here investigate the cues that parasites use to make their reproductive decisions by examining whether the gametocyte investment and sex ratio of a single clone infection change in response to material (‘cues’) derived from uninfected RBCs, RBCs infected with con-generic parasites, and RBCs infected with a con-specific genotype. The experiments were designed to build on previous work [16,25] to more specifically test ‘what’ parasites sense in their in-host environment. For example, in previous experiments conversion rates [16] and sex allocation [25] were compared in single and mixed genotype infections to ask whether parasites respond to in-host competition. However, numerous factors vary between single and mixed infections (e.g. anaemia, the age structure of RBCs, the concentration and balance of cytokines and the density of parasites) in complex ways. This makes it difficult to pinpoint exactly which factor(s) parasites are responding to. Furthermore, these changes in the in-host environment offer different opportunities and constraints to parasites that could be incorrectly interpreted as a parasite response. For example, parasites may not respond directly to anaemia, but may appear to do so, because a lack of preferred RBCs available for parasites to invade could directly interfere with their replication rate. The experiments presented here were designed to minimize the problem of simultaneously changing multiple aspects of the in-host environment, with the aim of getting closer to identifying the factor(s) which parasites are sensitive to.

## Methods

### Hosts and parasites

The rodent malaria parasite *P. chabaudi,* genotypes AJ and ER [53] were used. These wild-type clonal genotypes were originally isolated from areas where mixed infections were frequent [54]. Male MF1 mice, between ten and 12 weeks of age (in-house supplier, The University of Edinburgh), were kept in groups of two to five under a 12-hour light/dark cycle, at 21°C and provided *ad libitum* with food and water containing 0.05% para-aminobenzoic acid (PABA); a growth factor for parasites. Dynamics of the *P. chabaudi* AJ infections were monitored when exposed to treatments consisting of material derived from self, non-self (genotype ER), and RBCs (detailed below and in Table 1). AJ was chosen as the focal genotype, because it has been shown to respond to competition from unrelated strains with large changes in gametocyte investment and sex ratio [16,25]. All procedures were carried out in accordance with the UK Home Office regulations (Animals Scientific Procedures Act 1986) and approved by the ethical review panel at The University of Edinburgh.

**Table 1:** Summary of cue treatment groups, sample sizes, rationales, and classifications

**Cue treatment**	**N**	**Rationale**	**Classification**		
			**Treatment**	**Lysed parasites**	**Lysed RBC**
Control	5	No-treatment control for the stress of handling and injections	C	NP	NL
Uninfected RBC	5	Control for the stress of handling and injecting the host with blood	U	NP	NL
Uninfected lysed RBC	10	To test for a response to RBC debris	UL	NP	L
AJ-infected lysed RBC	10	Compare AJ to UL to test for a response to high density of self	AJ	P	L
ER-infected lysed RBC	10	Compare ER to AJ to test for a response to non-self	ER	P	L

The analysis involved comparing individual cue treatments and comparing treatments grouped in different ways to test whether parasites respond to lysed parasite material (P vs NP) and/or to lysed RBC material (L vs NL) . N = number of mice that received a particular treatment.

### Cue treatments

The experiment consisted of five treatment groups that received different cues injected into hosts (Table 1). The cue treatments, and the acronyms they are hereafter referred to as, are: (i) unmanipulated control, ‘C’; (ii) uninfected whole RBCs control, ‘U’; (iii) uninfected lysed RBCs, ‘UL’; (iv) AJ-infected lysed RBCs, ‘AJ’; and, (v) ER-infected lysed RBCs, ‘ER’. Note that these cues do not include the administration of additional live self (AJ) or competing (ER) parasites, nor do they directly affect the amount of RBC resources available to the focal AJ parasites. This avoids the potential problem of incorrectly interpreting a change in gametocyte investment or sex ratio as a parasite strategy when, for example, competition limits the availability of RBCs for gametocyte development, or induces immunity that increases gametocyte mortality.

The use of lysed *P. chabaudi* infected RBCs was inspired by recent demonstrations that asexual stages contain products that are packaged into ‘exosomes’ or ‘microvesicles’ to stimulate sexual differentiation in recipient parasites [55,56]. AJ infected RBCs (AJ) and ER infected RBCs (ER) were chosen to examine whether parasite products can be used to discriminate kin from non-kin (i.e., determine the presence of a con-specific genotype) in mixed infections, as suggested by previous experiments [16,25,30,57]. It is also possible that the high concentration of parasitized material in the AJ and ER cues mimicked a high density infection or high parasite mortality. Lysed, uninfected RBCs (UL) were intended to act as a control for the lysed, parasitized material, to distinguish whether any responses to the AJ and ER cues were due to parasite products or the lysed RBCs themselves. It is also possible that the administration of lysed uninfected RBCs mimics anaemia because many uninfected RBCs are lysed during an infection and gametocyte investment and sex ratio correlate with RBC resource availability [9,18,23]. Cells (RBCs and parasites) and the serum of the blood they were collected in were present in the cues. This was to maximize the chance that the cue material contained all potentially relevant factors, for example molecules released from inside cells, membrane components, or immune factors in the plasma.

To prepare the cue material, eight mice were infected via intraperitoneal (IP) injection with 1 × 10^6^ AJ parasitized RBCs, and eight separate mice with 1 × 10^6^ ER parasitized RBCs; both passaged from donor mice. When these infections reached their peak densities (on day 7 or 8 post infection (PI)), blood (infected with parasites at ring and trophozoite stages) was extracted from anaesthetized mice via cardiac puncture. Total blood volume, RBC density and parasite density were recorded for each mouse. The AJ and ER infected blood was pooled separately. The density of parasites in the pooled blood for each strain was similar; for AJ this was 1.61 × 10^9^ parasitized RBCs/ml of cue and for ER-infected blood this was 1.31 × 10^9^ parasitized RBCs/ml of cue. RBC densities were also similar, with an average RBC density for the AJ cue of 5.14 × 10^9^ RBCs/ml blood and 4.77 × 10^9^ RBCs/ml blood for the ER cue. Blood from naïve mice was collected for the UL cue. The RBC density for blood from naïve mice was much higher (9.06 × 10^9^ RBCs/ml blood) than for the AJ- and ER-infected mice. Therefore, to ensure RBC density was consistent across all cues, the blood for the UL cue was diluted with serum from uninfected mice, to give a final RBC density of 4.53 × 10^9^ RBCs/ml blood. For each of the cue treatment groups requiring lysed material (AJ, ER, UL) the cues went through four cycles of freeze–thaw, to ensure lysis of RBC and parasite membranes. Lysed cues were confirmed not to contain any live parasites capable of initiating an infection prior to the experiment, as follows. Three naïve mice each received 2 × 100 μl IP injections of the AJ cue with a four-hour gap between injections. PCR analysis of blood DNA samples [58] taken from the three mice confirmed that no parasite material was present in the blood 48 hours after injection of the cue and no infections appeared over the subsequent two weeks. Finally, for the U cue treatment group, blood was obtained via cardiac puncture from a naïve mouse immediately before it was injected as a cue.

On treatment days, 2 × 100 μl of cue material was administered to hosts via IP injection, with a four-hour gap between the injections. For the AJ cue, each host received a total of 1.03 × 10^9^ lysed RBCs, of which 3.21 × 10^8^ were parasitized. For the ER cue, each host received a total of 9.53 × 10^8^ RBCs, of which 2.62 × 10^8^ were parasitized. The lysed parasite material that was administered in both the AJ and ER cues was at least at the density that is typically observed at the peak of live AJ infections (assuming some cue material is cleared by innate immune factors before reaching the bloodstream). For example, the mean parasite density at the peak of infection for the control group, in cohort 2, of this experiment was 5.95 × 10^7^ parasites/ml blood. The cue administration regime (2 × 100 μl IP injections), with a four-hour gap between injections was chosen from pilot studies because it results in parasite material being detectable (by PCR) in the blood from 20 minutes and up to 24 hours post administration of the first cue; ensuring that cues are present in the bloodstream during the ring and trophozoite stages of the asexual cycle. Exposing a large proportion of the asexual cycle to cue treatments was necessary, because it is not known which stage is responsible for detecting the environmental signals that influence gametocyte investment and sex ratio decisions.

### Experimental design

Two cohorts, each containing 40 mice, were used to compare the effect of the cues administered during the pre-peak phase (day 4 PI; cohort 1) and post-peak phase (day 10 PI; cohort 2) of AJ focal infections (Table 1). Whilst transmission can occur throughout *P. chabaudi* infections, these time-points were chosen specifically because previous studies have revealed that this is when the largest effects of mixed-genotype infections on gametocyte investment and sex ratio have been observed [16,25]. On day 0, all mice were infected with 1 × 10^6^ AJ parasitized RBCs via IP injection, and mice were randomly allocated to the cohorts and cue treatment groups. Gametocyte density and sex ratio were examined on the days of cue administration to verify that there was no significant variation across treatment groups that could confound the detection of parasite responses. For *P. chabaudi*, it is thought that committed parasites differentiate into gametocytes in the cycle following the detection of a cue, that gametocytes require approximately 48 hours to reach maturity, and gametocytes remain infectious for a further 24 hours [33]. Therefore, to cover the period over which the focal AJ parasites could detect cues, adjust their reproductive strategies in response, and for the resulting gametocyte investment and sex ratios phenotypes to be detected, infections were monitored over the three days (i.e., three asexual cycles) following cue administration. To check whether aspects of the in-host environment (known to influence reproductive strategies, which could confound parasite responses to the cues given) varied across the treatment groups, the densities of RBCs, asexual stages and the proportion of RBCs that were reticulocytes were also monitored for three days post cue administration. The experiment was designed so that the responses to all cues could be compared to each other, and so that some cues could be combined to test for general responses to lysed parasites and/or lysed RBCs by grouping cue treatments into those containing parasite material (‘P’) or not (‘NP’), and those containing lysed RBC material (‘L’) or not (‘NL’), (Table 1).

### Data collection and analysis

Blood samples (taken from tail snips) were collected for thin smears (to count reticulocyte proportion), to measure RBC densities (using flow cytometry, Beckmann Coulter counter), and for DNA and RNA to quantify parasites, gametocytes and sex ratios. Samples were collected daily, from day 2 to day 15 PI for both cohorts, but analyses were restricted to day 4 to day 7 PI for cohort 1, and day 10 to day 14 PI for cohort 2. Mouse weight was monitored every other day for both cohorts. All samples were obtained in the morning when parasites were at ring stage, before DNA replication for the production of daughter progeny had occurred. The density of reticulocytes was calculated from examination of blood smears and coulter count readings. DNA and RNA were extracted from blood samples using the ABI Prism 6100 Nucleic Acid PrepStation and the Bloodprep chemistry (for DNA, Life Technologies) or total RNA chemistry system (RNA, LifeTechnologies) as described in [58]. cDNA was generated from RNA and quantitative PCR was used to quantify DNA or cDNA, according to the protocols outlined in [58]. Real-time PCR was performed a) on DNA using CG2 primer pairs [30] to quantify asexual parasites, b) on cDNA using CG2 primer pairs to quantify total gametocytes, and, c) on cDNA using MG8 primer pairs to quantify male gametocytes, according to the protocols outlined in [58]. Sex ratios were calculated by dividing the number of male gametocytes by the total number of gametocytes in any given sample.

Data were analysed using R version 3.0.2 [59]. Response variables were log transformed (gametocyte density) or arcsine square root transformed (sex ratio) to meet the assumptions of normality. ANOVAs were performed to compare RBC densities, reticulocyte densities and asexual densities across cue treatment groups. Comparisons were made on the day of cue administration before cues were given, and for the following three days. The cumulative gametocyte densities for three days post cue administration were used to compare gametocyte investment decisions across treatments. In this case, it was appropriate to use gametocyte density as a measure of gametocyte investment because asexual densities did not vary significantly across the treatment groups before cue administration (see Table 2). This means that any observed differences in gametocyte density must result from different levels of gametocyte investment (i.e., given that all else is equal, variation in gametocyte densities can only result from variation in investment in response to cues). This approach also avoids the difficulties of accurately calculating gametocyte investment [21], especially when the time period between parasites detecting cues and their response being measurable is uncertain. Similarly, for sex ratio, the time between parasites detecting cues and their response being measurable is uncertain, so the mean sex ratio for the three days post cue administration was compared across groups. Finally, Welch’s T test was used to compare the effects of parasitized *versus* non-parasitized cues and lysed *versus* non-lysed cues on cumulative gametocyte densities and mean sex ratios for both cohorts 1 and 2. The number of samples analysed varied between tests because (a) some mice died during the experiment, and (b) total and male gametocyte densities below the lower limits of detection for the PCR were excluded, because quantification was unreliable.

**Table 2:** Summary of ANOVA analyses

	**Cohort 1**		**Cohort 2**	
	**Prior: day 4**	**Post: days 5-7**	**Prior: day 10**	**Post: days 11-13**
**Asexual density**	F_4, 34_ = 1.13, p = 0.36	F_4, 34_ = 0.79, p =0.54	F_4, 25_ = 0.59, p = 0.68	F_4, 24_ = 0.14, p = 0.97
**RBC density**	F_4, 34_ = 1.00, p = 0.42	F_4, 34_ = 1.70, p = 0.17	F_4, 28_ = 1.62, p = 0.20	F_4, 24_ = 0.45, p = 0.77
**Reticulocyte proportion**	F_4, 34_ = 1.05, p = 0.40	F_4, 34_ = 0.32, p =0.86	F_4, 28_ = 0.77, p = 0.56	F_4, 24_ = 1.53, p = 0.23
**Gametocyte density**	F_4, 34_ = 0.17, p = 0.95	F_4, 34_ = 0.39, p = 0.81	F_4, 28_ = 1.60, p = 0.20	F_4, 20_ = 1.73, p = 0.18
**Sex ratio**	F_4, 31_ = 1.27, p = 0.30	F_4, 34_ = 0.60, p = 0.67	F_4, 28_ = 0.63, p = 0.64	F_4, 26_ = 0.22, p = 0.93

Asexual density and the in-host environmental parameters of RBC density and proportion of reticulocytes did not vary significantly across the treatment groups - either prior to, or post cue administration, in either cohort. Furthermore, gametocyte density and sex ratio did not vary significantly prior to cue administration. This means that the effects of the cue treatments were not confounded by unintended variation in the in-host environment or pre-existing variation in gametocyte density and sex ratio (see also Additional file 1: Figure S1).

## Results

### Asexual densities and in-host environmental variables

Asexual density, RBC density, and the proportion of RBCs that are reticulocytes all correlate with reproductive decisions and so variation in these parameters across treatment groups could confound any responses to the cue treatments. However, there was no significant variation in these parameters across treatment groups, either before cue administration, or over the subsequent three-day period, for either cohort (Table 2, Additional file 1: Figure S1).

### Gametocyte investment

**Figure 1:**
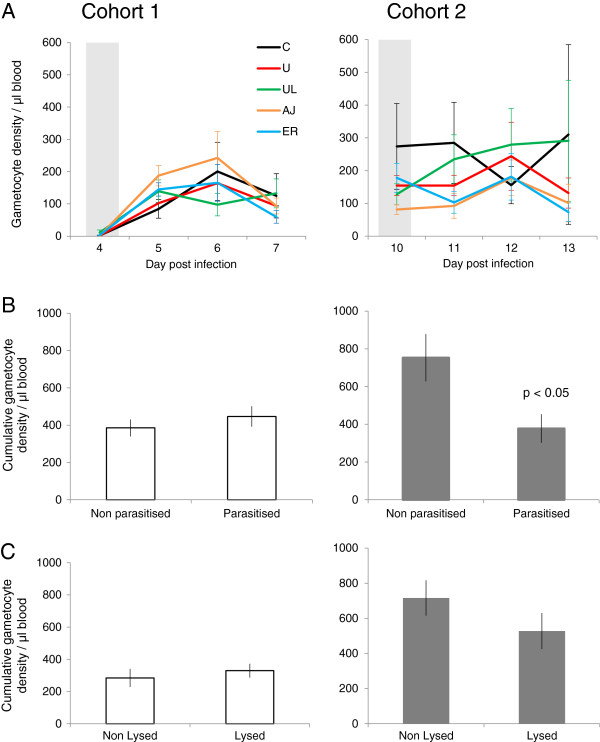
***Plasmodium chabaudi *AJ gametocyte density dynamics.** (± SEM) from the day of administration of five cue treatments: C: control, U: uninfected RBCs, UL: uninfected lysed RBCs, AJ: AJ infected lysed RBCs and ER: ER infected lysed RBCs). Grey bars indicate the days when cues were administered - on day 4 PI for cohort 1 (left) and day 10 PI for cohort 2 (right) **(A)**; cumulative gametocyte densities (± SEM) for three days post treatment with cues containing parasitized material (P: AJ, ER) or non-parasitized material (NP: C, U, UL) for cohort 1 (left) and for cohort 2 (right: where gametocyte density was significantly lower in the P group than NP group) **(B)**; cumulative gametocyte densities (± SEM) for three days post treatment with either lysed RBC material (L: UL, AJ, ER) or non-lysed material (NL: C, U) for cohort 1 (left) and cohort 2 (right) **(C)**.

Gametocyte densities were not significantly different between treatment groups either pre peak of infection (cohort 1) or post peak (cohort 2) on the days of cue administration (Figure 1A and Table 2). This result, together with the validation that asexual densities and in-host environmental variables were not significantly different prior to cue administration means that, in this study: gametocyte density is synonymous with gametocyte investment. For the three days following cue administration, there were no significant differences in cumulative gametocyte densities between the five cue treatment groups in either cohort 1 or cohort 2 (Figure 1A and Table 2). When treatments were grouped to compare the effect of cues containing parasitized (P) *versus* non-parasitized (NP) material, there were no significant differences in gametocyte densities in cohort 1 (t (35.8) = 0.83, p = 0.41) (Figure 1B)*.* However, in cohort 2, gametocyte density was significantly 50% lower in infections that received parasitized cues (378 ± 75 gametocytes/μl blood), compared to those that received non-parasitized cues (753 ± 125 gametocytes/μl blood), (t (22.9) = −2.19, p = 0.04) (Figure 1B). Finally, when treatments were grouped to compare cues containing lysed (L) or non-lysed (NL) material, there were no significant differences for cohort 1 (t (12.8) = 0.12, p = 0.91) or cohort 2 (t (6.6) = −1.47, p = 0.19) (Figure 1C).

### Sex ratio

**Figure 2:**
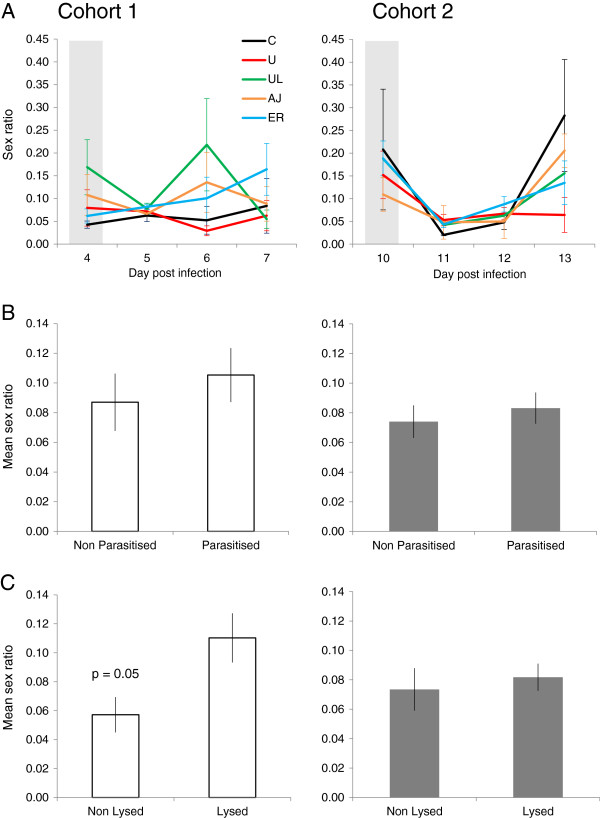
***Plasmodium chabaudi *AJ sex ratio (proportion of male gametocytes) dynamics.** (± SEM) from the day of administration of five cue treatments: C: control, U: uninfected RBCs, UL: uninfected lysed RBCs, AJ: AJ-infected lysed RBCs and ER: ER-infected lysed RBCs. Grey bars indicate the days when cues were administered - on day 4 PI for cohort 1 (left) and day 10 PI for cohort 2 (right) **(A)**; mean sex ratio (± SEM) for three days post treatment with cues containing parasitized material (P: AJ, ER) or non-parasitized material (NP: C, U, UL) for cohort 1 (left) and cohort 2 (right) **(B)**; mean sex ratio (± SEM) for three days post treatment with either lysed RBC material (L: UL, AJ, ER) or non-lysed material (NL: C, U) for cohort 1 (left: where sex ratio was significantly (borderline) higher in the lysed group), and for cohort 2 (right) **(C)**.

Sex ratios (proportion of male gametocytes; Figure 2A) were not significantly different between cue treatment groups for cohort 1 or cohort 2 on the days of cue administration (Table 2). Therefore, as for gametocyte density, there was no pre-existing significant variation in sex ratios that could have confounded any changes in sex ratio following the cue treatments. For the three days following cue administration there were no significant differences in mean sex ratios between the five treatment groups in cohort 1 or cohort 2 (Figure 2A and Table 2). When cue treatments were grouped to compare the effect of parasitized (P) *versus* non-parasitized (NP) material, there were no significant differences in mean sex ratio in cohort 1 (t (36.7) = 0.66, p = 0.51), or in cohort 2 (t (27.8) = −0.35, p = 0.73) (Figure 2B). However, when treatments were grouped to compare the effects of cues containing lysed (L) or non-lysed (NL) material, there was a borderline significant increase in sex ratio (of 45%) in infections that received lysed material (0.11 ± 0.02), compared to those that received non-lysed cues (0.06 ± 0.01) in cohort 1 (t (27.0) = 2.04, p = 0.05), but not in cohort 2 (t (9.87) = −0.13, p = 0.90) (Figure 2C).

## Discussion

The experiments presented here reveal that: (i) gametocyte investment is reduced by 50% in response to lysed material containing parasites (P) compared to material without parasites (NP); (ii) the change in gametocyte investment in response to parasitized material occurs post peak of infections, but not during the growth phase; (iii) there was a borderline significant increase (45%) in the proportion of male gametocytes in infections given lysed (L) compared to non-lysed (NL) material; and, (iv) the potential sex ratio adjustment in response to lysed material only occurred in the growth phase of infections. The following paragraphs discuss how these results compare to studies of human and rodent infections that report changes in sex ratio and gametocyte investment in response to variation in RBC resource availability, drugs, competition, and parasite density [11-16,18,23-25,29,31-34].

In the post-peak phase of infections, why do parasites make different gametocyte investment decisions when exposed to material derived from non-parasitized (NP) blood compared to parasitized blood (P, Figure 1B)? Gametocyte investment is lower in the P group compared to NP group which suggests that either the parasites in the P group are adopting reproductive restraint (i.e., actively reducing investment) or the parasites in the NP group are making a terminal investment (i.e., actively increasing investment). The former scenario is the most likely for the following reasons. When parasites are faced with adverse, but not lethal, circumstances either due to resource limitation or death rates that do not exceed the capacity for replication, they are predicted to adopt a strategy of reproductive restraint [19,21,39]. Lysed parasite material in the P group could signal that many parasites are being killed (e.g., due to immune attack or drugs) and reproductive restraint enables the replication rate to exceed the death rate. The ability to predict future scenarios may seem highly sophisticated for parasites, but this is one of the main evolutionary drivers of adaptive phenotypic plasticity [60,61]. Preparing for environmental change in advance avoids fitness costs incurred by delays involved in waiting for the environment to change and then reacting, or not reacting to environmental change at all [62]. Second, the gametocyte investment of parasites in the NP group appears too low to be explained by terminal investment. This is because the NP group includes the unmanipulated control group and most studies use such infections as a baseline to demonstrate that increased investment (i.e., terminal investment) occurs in response to drugs. In summary, gametocyte investment appears to be reduced in response to material from parasitized blood, which is consistent with parasites adopting reproductive restraint to maximize survival during stressful, but not lethal, challenges during infections [14,16,30].

Instead of parasites actively adjusting gametocyte investment, could differential immune responses in the P and NP groups explain the observed differences in gametocyte investment? It is possible that the administration of lysed parasitized material induced the host to produce the pro-inflammatory cytokines interferon gamma (IFN-gamma) and tumour necrosis factor (TNF), which are known to be involved in killing gametocytes [63-65]. However, data from *in vitro* studies suggest this would be unlikely, as the induction of TNF and IFN-gamma is much reduced when exposed to lysed parasitized RBCs, compared with exposure to live intact parasitized RBCs [66-69]. Furthermore, the induction of TNF by lysed parasites in culture is negligible when the parasitized erythrocytes were harvested and lysed at ring and/or trophozoite stages (compared to lysis at schizont stage) [68]. As such, the P group (a lysed mixture of ring and trophozoite infected erythrocytes) is unlikely to have induced TNF to a level that was sufficient to clear gametocytes. Furthermore, the gametocytocidal activity of TNF is rapid [63] and would, therefore, have produced a sharp drop in the P group on day 11 only, which was not observed. Finally, the cue treatments were the same in cohort 1 and 2 and so should elicit the same immune responses. If these responses killed gametocytes then fewer gametocytes would have been observed in the P group of cohort 1 as well, but this was not the case.

The question of why parasites only adopted reproductive restraint in response to parasite material in the post-peak phase (i.e., in cohort 2) of infections requires further work. This timing is consistent with previous studies showing that the difference in gametocyte investment between parasites in control and sub-lethal conditions increases over time [14,16]. Furthermore, the timing suggests a biologically significant difference in phenotype with real epidemiological relevance, as it is at this later stage of *P. chabaudi* infections where transmission is typically most successful in laboratory studies [70]. Furthermore, a twofold reduction in gametocyte density in *P. falciparum* infections can have a significant impact on the proportion of mosquitoes infected [71]. The lack of any effect in the pre peak phase of the infection may be due to the difficulty in detecting small effects at low parasite densities (as is the case early in infections), or because parasites become increasingly able to detect, or respond to, environmental changes as infections progress. The latter is perhaps the most parsimonious explanation because cumulative gametocyte densities are very similar between all of cohort 1 and the P group of cohort 2 (Figure 1B; (t (41.5) = −1.02, p = 0.31). This may reflect a necessity to maintain a baseline level of gametocyte production to ensure no transmission opportunity is wasted, even during reproductive restraint.

Why might parasites make different sex ratio decisions when exposed to material derived from lysed cells (L; parasites and RBCs), and why is this only observed in the growth phase of infections? Further work is required to confirm whether parasites do produce a less female-biased sex ratio when exposed to lysed cues (because significance was borderline), but this pattern is predicted by evolutionary theory and consistent with other data [25,46,72]. Lysed material could either represent host anaemia, or the material could have stimulated innate host immune responses that reduce the fertility of males more than females. In these situations, males become a limiting resource for fertilization and so parasites are predicted to partially compensate by increasing their investment in male relative to female gametocytes [25,46,47,72-74]. That extra males are required to ensure females are fertilized when transmission blocking immune factors have more severe effects on males is intuitive, but why are more males required when hosts are anaemic? Each male gametocyte can produce up to eight gametes, but each female only produces one gamete, which means that the number of parasite progeny is maximized at a ratio of eight female gametocytes to one male gametocyte [6,44]. However, when there are eight-fold fewer male gametocytes circulating in the host and gametocyte density is very low, or hosts are anaemic, there is a stochastic risk that blood meals do not contain enough males to ensure the females are fertilized [46,72]. Therefore, if lysed material represents anaemia and/or immune factors, parasites will be most sensitive to these scenarios when gametocyte density is low (i.e., in cohort 1; Figure 1C). In summary, similarly to the gametocyte investment results, the sex ratio data suggest lysed cell material (parasitized and non-parasitized) is interpreted as a cue for adverse conditions.

Based on previous observations of mixed genotype infections and evolutionary theory [6,15-17,25,39,44,75], parasites were predicted to adopt different reproductive strategies when exposed to cue material derived from self (AJ) *versus* a non-self, con-specific genotype (ER). However, there were no significant differences either in gametocyte investment (Figure 1A) or sex ratio (Figure 2A) when parasites were exposed to AJ *versus* ER cue material, in either cohort. This could be due to a number of (non-mutually exclusive) reasons. First, there may not have been a high enough concentration of lysed ER parasite material in the bloodstream in the ER group for live AJ parasites to discriminate kin from non-kin. Alternatively, the cue to discriminate kin may be something that is only actively secreted by live parasites in direct response to competitors (which were not present in the cue-generating infections), or degraded in the freeze-thaw process. For example, malaria parasites could employ a similar quorum-sensing strategy to that observed in bacteria [76,77] and use microvesicles [56] or exosome-like vesicles [55] derived from infected RBCs as a carrier for the cue. However, microvesicle or exosome structures may have been destroyed during cue preparation lysis. The cue treatments were designed simply to test whether parasite responses could be elicited, rather than to identify precisely what they are detecting, so it is possible that the live AJ parasites could discriminate kin, but the AJ and ER cues also represented other scenarios (e.g., a high death rate), that provided a stronger stimulus and resulted in the responses detected.

## Conclusions

Despite decades of investigating gametocytes, how the genes and molecular pathways underpinning commitment to gametocytes and sexual differentiation interact with environmental sensing has proved elusive [2,3,78], although recent characterization of the ApiAP2 gene in *P. falciparum* [UniProt:PFL1085w/PF3D7_1222600] and *Plasmodium berghei* [PlasmoDB: PBANKA_143750] is promising [79,80]. The difficulty may be partly due to different genes and pathways being involved in: (a) sensing environmental cues relevant to decisions about reproductive strategies; (b) processing information and making decisions; and, (c) producing the gametocyte investment and sex ratio phenotypes resulting from the decisions made [21]. Breaking down treatments to isolate the molecule(s) used as a cue(s) within the morass of lysed cells and serum used in this study could facilitate further characterization of molecular mechanisms underpinning commitment and differentiation into gametocytes. Repeating the experiments presented here *in vitro,* to expose synchronous parasites at different time points within the cell cycle could reveal which developmental stages are responsible for sensing and responding to changes in the in-host environment. More broadly, it may be possible to harness cues to ‘trick’ parasites in an infection into producing gametocytes instead of asexuals, or only producing gametocytes of a single sex [21,81]. The former strategy could be useful for treating returned travellers in hospital (without malaria vectors) because the virulence of infections will be reduced, and the latter strategy would prevent fertilization and subsequent transmission. Finally, precisely identifying the cues that parasites use to make reproductive decisions is required to quantify the costs and benefits (fitness consequences) of their strategies, which is central to understanding their evolution.

## Abbreviations

RBC: Red blood cell; TBI: Transmission blocking immunity; PI: Post infection; IP: Intraperitoneal; C: Control cue treatment; U: Uninfected whole RBC cue treatment; UL: Uninfected lysed RBC cue treatment; AJ: Lysed AJ-infected cue treatment; ER: Lysed ER-infected cue treatment; P: Parasitized material group; NP: Non-parasitized material group; L: Lysed material group; NL: Non-lysed material group.

## Competing interests

The authors have declared that they have no competing interests.

## Authors’ contributions

LMC and SER designed the experiments, LMC and PS performed the experiments, LMC and SER analysed the data and all authors contributed to the manuscript. All authors read and approved the final manuscript.

## Supplementary Material

Additional file 1: Figure S1 *Plasmodium chabaudi* AJ infection dynamics: mean (± SEM) for each cue treatment (C: control, U: uninfected RBCs, UL: uninfected lysed RBCs, AJ: AJ-infected lysed RBCs and ER: ER-infected lysed RBCs) administered on day 4 PI for cohort 1 (left) and day 10 PI for cohort 2 (right) (indicated by grey bars). RBC density dynamics (A); proportion of RBCs that are reticulocytes (B) and asexual density dynamics (C). Maximum values for the Y axes differ between cohort 1 and cohort 2 to allow clear visualization of the range of data for each cue treatment group. Click here for file
