# Supplementary material for: Information use and plasticity in the reproductive decisions of malaria parasites
Source: Malar J. 2014 Mar 26;13:115. doi: 10.1186/1475-2875-13-115 (PMC3986881; doi:10.1186/1475-2875-13-115)
Supplement: Additional file 1: Figure S1 — Plasmodium chabaudi AJ infection dynamics: mean (± SEM) for each cue treatment (C: control, U: uninfected RBCs, UL: uninfected lysed RBCs, AJ: AJ-infected lysed RBCs and ER: ER-infected lysed RBCs) administered on day 4 PI for cohort 1 (left) and day 10 PI for cohort 2 (right) (indicated by grey bars). RBC density dynamics (A); proportion of RBCs that are reticulocytes (B) and asexual density dynamics (C). Maximum values for the Y axes differ between cohort 1 and cohort 2 to allow clear visualization of the range of data for each cue treatment group. [file 1475-2875-13-115-S1.pptx]

## Slide 1
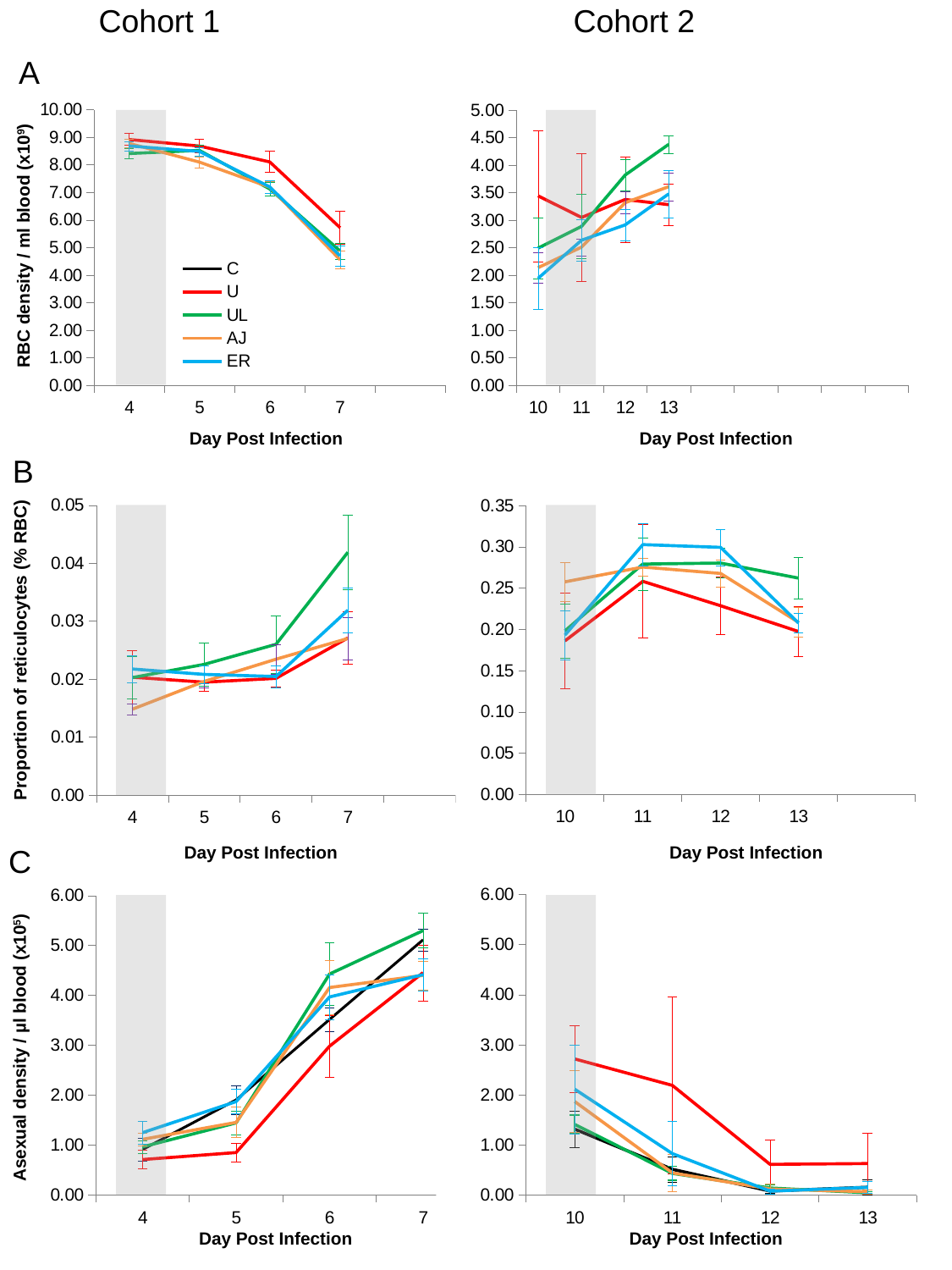

Cohort 1
Cohort 2
A
### Chart
| Category | C | U | UL | AJ | ER |
|---|---|---|---|---|---|
| 4 | 8.617600000000001 | 8.918000000000001 | 8.408800000000001 | 8.775222222222222 | 8.683000000000002 |
| 5 | 8.481000000000003 | 8.680200000000001 | 8.521899999999999 | 8.097555555555555 | 8.4844 |
| 6 | 7.815999999999998 | 8.104000000000001 | 7.123499999999996 | 7.1887777777777755 | 7.196499999999999 |
| 7 | 5.9374 | 5.7202 | 4.8768 | 4.556222222222223 | 4.6983999999999995 |
### Chart
| Category | | | | | |
|---|---|---|---|---|---|
| 10 | 2.79475 | 3.4415999999999998 | 2.4906249999999996 | 2.1363749999999997 | 1.9415 |
| 11 | 2.9106666666666654 | 3.049 | 2.8875 | 2.5106249999999997 | 2.635428571428572 |
| 12 | 3.195 | 3.3759999999999994 | 3.820285714285714 | 3.317142857142858 | 2.9135714285714305 |
| 13 | 3.710666666666667 | 3.2816 | 4.3787142857142864 | 3.608999999999999 | 3.4782857142857138 |
RBC density / ml blood (x109)
### Chart
| Category | C | U | UL | AJ | ER |
|---|---|---|---|---|---|
| 4 | 0.016104000000000004 | 0.020334 | 0.020264 | 0.0148 | 0.021750000000000002 |
| 5 | 0.0163374 | 0.019470200000000003 | 0.022539900000000005 | 0.019598700000000004 | 0.0208117 |
| 6 | 0.0190926 | 0.0201294 | 0.026011500000000003 | 0.0234396 | 0.020449100000000005 |
| 7 | 0.027359666666666702 | 0.027118 | 0.0419116666666667 | 0.027021011111111107 | 0.03191677777777781 |Day Post Infection
Day Post Infection
B
### Chart
| Category | | | | | |
|---|---|---|---|---|---|
| 10 | 0.23015 | 0.18608000000000002 | 0.19825 | 0.25763749999999996 | 0.1931 |
| 11 | 0.2909000000000001 | 0.25820000000000004 | 0.279175 | 0.2755125000000001 | 0.30272857142857107 |
| 12 | 0.2955333333333329 | 0.22866 | 0.2803571428571431 | 0.2678 | 0.29938571428571403 |
| 13 | 0.1708 | 0.19744000000000003 | 0.26214285714285707 | 0.208642857142857 | 0.20770000000000002 |
Proportion of reticulocytes (% RBC)
C
Day Post Infection
### Chart
| Category | C | U | UL | AJ | ER |
|---|---|---|---|---|---|
| 10 | 1.3162341193199159 | 2.7160325465965274 | 1.4092702753841866 | 1.8666058296032255 | 2.1125127346856245 |
| 11 | 0.5152802085876459 | 2.1883774651527412 | 0.433399536526203 | 0.443441834193468 | 0.828142232497249 |
| 12 | 0.081182424736023 | 0.6135299127221111 | 0.141957070499659 | 0.12170154173033602 | 0.07962597459724971 |
| 13 | 0.15688629293839101 | 0.6296161238729949 | 0.046550383168458886 | 0.06770962889705391 | 0.15429535957404505 |Day Post Infection
Day Post Infection
### Chart
| Category | C | U | UL | AJ | ER | |
|---|---|---|---|---|---|---|
| 4 | 0.9107213214138521 | 0.7097502604156731 | 0.964175725432105 | 1.114231958715245 | 1.248489510119259 | None |
| 5 | 1.9054005060768133 | 0.849650986956954 | 1.4437113487795976 | 1.4543608073234258 | 1.870952276239097 | None |
| 6 | 3.5180559870621555 | 2.982491366941065 | 4.429038942183041 | 4.158379680249323 | 3.9686392117526683 | None |
| 7 | 5.114210974843612 | 4.455876163071006 | 5.29777108953101 | 4.400596751007126 | 4.41525185527772 | None |
Asexual density / µl blood (x105)
Day Post Infection
Day Post Infection
Day Post Infection
Day Post Infection
